# ADRENAL CORTICAL CARCINOMA IN INFANCY

**DOI:** 10.1590/1984-0462/;2019;37;1;00021

**Published:** 2019

**Authors:** Carlos Alberto Longui

**Affiliations:** a Faculdade de Ciências Médicas, Santa Casa de São Paulo, São Paulo, SP, Brazil.

A drenocortical carcinoma (ACC) in infancy is a serious disease of poor prognosis if the diagnosis is not promptly recognized. Surgical treatment with complete tumor removal is the main goal of therapy. The pediatrician plays an important role in the early detection of ACC by recognizing the major signs and symptoms of excessive production of androgens and glucocorticoids.

In Brazil, especially in the South and Southeast regions, the increased prevalence of *P53* gene mutation results in a 15 times greater frequency of ACC.^1^ Thus, pediatricians should be alert to the early onset (girls <8 years; boys <9 years) of signs, such as growth spurt, bone age advancement, axillary sweating and odor, increase in the oiliness of the skin, appearance of acne, and onset of secondary sexual characteristics, e.g., pubic hair, darkening of genital skin, and growth in the penile or clitoral corpus cavernosum. Excessive glucocorticoid action (Cushing’s syndrome) can also be present.

Early androgenic signs are common in other less serious conditions, which correspond to the main differential diagnoses of ACC. Idiopathic premature adrenarche (early onset of adrenal androgen production) is the most frequent benign condition, especially in premature patients, those small for their gestational age, or conceived by *in vitro* fertilization. Congenital adrenal hyperplasia is another relatively common condition.

Faced with a clinical suspicion of ACC, it is mandatory that the pediatrician proceeds to an image investigation of the adrenal glands. Computed tomography is the examination with better detection rate, best cost-benefit ratio, and lower false-positives and negatives rates. Adrenal ultrasonography requires an experienced professional and possible false-negative result in small tumors.

Even in the presence of positive adrenal imaging, it is essential to investigate the basal adrenal steroid production in order to recognize a non-functioning incidentaloma. This is performed by the quantitation of dehydroepiandrosterone sulfate (DHEAS), 17-hydroxyprogesterone (17OHP), 11-deoxycortisol (compound S), androstenedione (A4), testosterone, and cortisol. Another fundamental step is to demonstrate that the adrenal steroid production is autonomous by conducting the dexamethasone suppression test. In our Unit, we perform the suppression test with 3.75 mg/m^2^ of dexamethasone, divided into four daily doses (6 a.m./noon/6 p.m./midnight) for five consecutive days. The final sample collection is obtained at 8 a.m. (2 hours after the last dose of the test).

Indications of increased risk for ACC are early androgenic signs, fast progression, combination of virilization and Cushing signs, family history of adrenal tumor, and association with other tumors.

The differentiation between adrenocortical carcinoma and adenoma in clinical practice is still difficult. Several studies have pointed out some clinical, anatomopathological, and molecular characteristics as potential markers of progressive malignancy.^2^ ^,^ ^3^

The study by Monteiro et al.^4^ reported in this issue of *Revista Paulista de Pediatria* brings the experience of a pediatric oncology group working at a teaching hospital in the state of Minas Gerais. They present and discuss demographic, hormonal, immunohistochemical, imaging, and clinical follow-up data, comparing them with what was previously described in the literature. The study emphasizes the importance that pediatricians must increase their ACC suspicion, as well as the need for promptness in referring the patient to a center with a specialized team. These measures are crucial for fast diagnosis and to decrease the interval between detection and surgery.
